# p53 Family and Cellular Stress Responses in Cancer

**DOI:** 10.3389/fonc.2014.00285

**Published:** 2014-10-21

**Authors:** Johanna Pflaum, Sophie Schlosser, Martina Müller

**Affiliations:** ^1^Department of Internal Medicine I, University Hospital Regensburg, Regensburg, Germany

**Keywords:** p53, p63, p73, cellular stress, cancer, chemosensitivity, apoptosis

## Abstract

p53 is an important tumor suppressor gene, which is stimulated by cellular stress like ionizing radiation, hypoxia, carcinogens, and oxidative stress. Upon activation, p53 leads to cell-cycle arrest and promotes DNA repair or induces apoptosis via several pathways. p63 and p73 are structural homologs of p53 that can act similarly to the protein and also hold functions distinct from p53. Today more than 40 different isoforms of the p53 family members are known. They result from transcription via different promoters and alternative splicing. Some isoforms have carcinogenic properties and mediate resistance to chemotherapy. Therefore, expression patterns of the p53 family genes can offer prognostic information in several malignant tumors. Furthermore, the p53 family constitutes a potential target for cancer therapy. Small molecules (e.g., Nutlins, RITA, PRIMA-1, and MIRA-1 among others) have been objects of intense research interest in recent years. They restore pro-apoptotic wild-type p53 function and were shown to break chemotherapeutic resistance. Due to p53 family interactions small molecules also influence p63 and p73 activity. Thus, the members of the p53 family are key players in the cellular stress response in cancer and are expected to grow in importance as therapeutic targets.

## Introduction

Human cells are constantly exposed to external and internal stressors, which cause damage to the integrity of the cell and to its genome. In order to guarantee the survival of the organism, cells have developed numerous strategies to adapt to stressors. In this review, we would like to discuss the influence of cellular stress on tumor development as well as strategies in cancer therapy targeting pathways involved in cell-cycle control and apoptosis. Special emphasis is put on the members of the p53 family.

## Cellular Stress Response in Cancer Development

The development of cancer is a multistep process that involves a series of mutations in the progenitor cell (1). It enables clonal proliferation, uncontrolled growth, and finally invasion (2, 3). Cellular stress can be caused by a multitude of external or internal influences such as ultraviolet radiation (4–6), ionizing radiation (7), hypoxia (8), carcinogens (e.g., aflatoxin) (9, 10), cigarette smoke (11), oxidative stress (12–14), and oncogene activation (15). This can lead to DNA damage and, in consequence, to malignant transformation of the cell. In order to restore its integrity, the cell disposes of a number of damage control mechanisms. These mechanisms are older than the human species and can already be found 1 billion years ago in descendants of choanoflagellates and the early metazoan sea anemone (16). Human tumor protein p53, often described as the “guardian of the genome,” and its target genes play key roles in cell-cycle control and induction of apoptosis. In its capacity as tumor suppressor protein, p53 is not only able to act as transcription factor for genes of pro-apoptotic effector proteins but it is also involved in transcription-independent cellular signaling leading directly to cell death via pathways originating from the mitochondria or the cytosol (17–19). Furthermore, p53 induces transcription of DNA repair enzymes, thereby promoting cell survival (20–22). This shows the functional dichotomy of p53. To date, the exact mechanisms deciding about death or survival of the damaged cell still remain to be elucidated. Under physiological conditions, cellular p53 levels are low and the protein has a relatively short-half-life of 20 min. Upon DNA damage, p53 levels rise primarily through stabilization of the protein (23).

**Figure 1:**
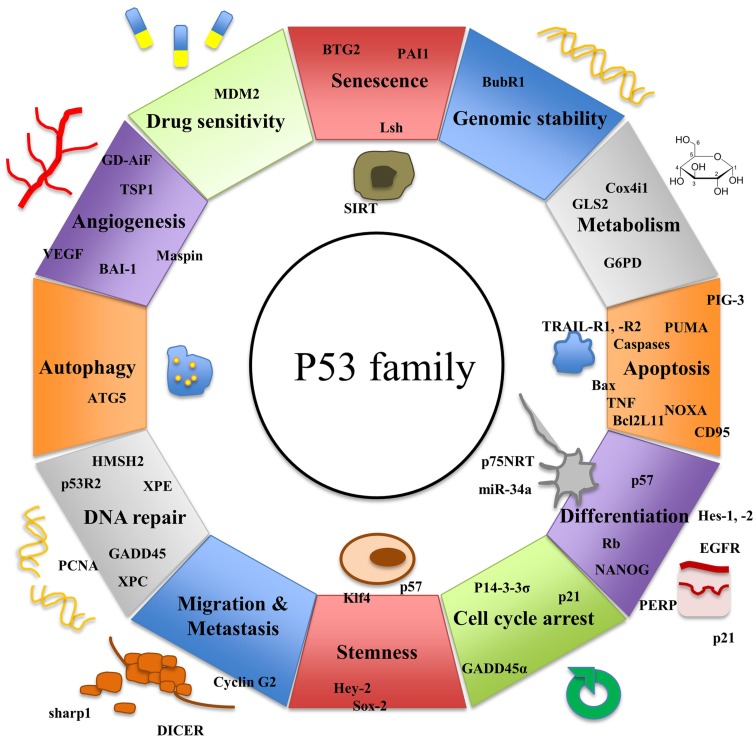
**Functions of p53 and its homologs p63 and p73 and their target genes**.

While p53 has been known for more than three decades, two further members of the p53 family, p63 and p73, have been discovered more recently. The three genes exhibit a high degree of homology and there is increasing evidence that they have risen from the triplication of a common ancestral gene (24, 25). All three genes consist of important structural elements including a DNA-binding domain (DBD), an oligomerization domain (OD), and a transactivation domain (TAD) (26). p63 (27, 28) and p73 (29) have been shown to induce apoptosis similarly to p53 via activation of several of its downstream target genes (30–32). Yet, both family members also exhibit functions distinct from p53 (Figure 1).

While p63 is crucially involved in craniofacial, limb, and skin development (33), p73 plays an important role during neurogenesis (34). Multiple isoforms of the p53 family members are generated using different promoters and alternative splicing. They can carry out contrary functions. Whereas some isoforms have oncogenic potential, others can act as tumor suppressors (35). However, many isoforms seem to have both capacities depending on the entity of the cell they are expressed in and the tissue context. To date, regulation and interactions of the three members of the p53 family are still under investigation.

## Apoptosis

Malignant tumors often exhibit defects in apoptosis signaling pathways, resulting in tumor cell survival. Therefore, understanding the exact mechanisms of apoptosis can provide new strategies for the development of anti-cancer treatments. The extrinsic apoptosis signaling pathway is initiated by ligands such as TNFα, CD95L, and TRAIL binding to death receptors (36–38). The best characterized members of the death receptor family are TNFR1, CD95, DR3, TRAIL-R1 (CD4), TRAIL-R2 (CD5), and DR6 (39, 40).

Death receptor signaling leads to activation of caspases. Caspases are cysteinyl aspartate proteinases, which are synthesized as inactive zymogens and, upon stimulation, are initialized by autolytic cleavage (41). Initiator caspases, such as caspase 8 und 9, form signaling complexes, which activate downstream effector caspases, including caspase 3 and 7, through proteolytic cleavage (41, 42). Effector caspases cannot self-activate but process a multitude of cellular substrates during cell death (43). The intrinsic apoptosis signaling pathway originates in the mitochondria and is part of the cellular stress response. It is regulated by proteins of the Bcl-2 family. Pro-apoptotic members of the protein family include Bax, Bak, and their subclass of BH-3 only proteins such as BAD, BID, BIM, Hrk, PUMA, BMF, and Noxa, whereas A1, Bcl-2, Bcl-w, Bcl-XL, and Mcl-1 are among the anti-apoptotic members (44). The anti-apoptotic Bcl-2 proteins exert their function by stabilizing the outer mitochondrial membrane (45). Upon cellular stress, Bid and Bim mediate homo-oligomerization of Bax and Bak, which leads to the release of cytochrome *c* from the mitochondrial intermembrane space (46). By binding Bcl-2 proteins Bad, Noxa, and PUMA lead to inhibition of the proteins (44). Being released into the cytosol, cytochrome *c* forms a complex with APAF-1 and pro-caspase 9. After cleavage, caspase 9 activates effector caspase 3 (44).

## p53 and Its Isoforms

**Figure 2:**
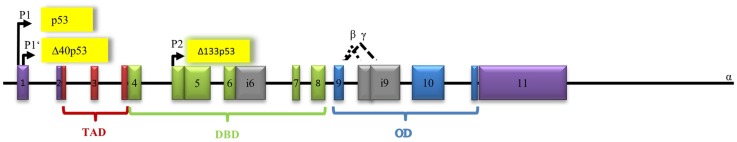
**Architecture of the human p53 gene structure: alternative splicing (α, β, γ), alternative promoters (P1, P1′, P2), transactivation domain (TAD), DNA-binding domain (DBD), and oligomerization domain (OD) are indicated**. The P1 promoter generates full-length-proteins with a transactivation domain (TAD), whereas the P1′- and P2 promoters generate proteins lacking the TAD.

p53 is encoded by the TP53 gene on the short arm of chromosome 17 and has a molecular mass of 43.7 kDa (25). It spans 19,200 bp including 11 exons (Figure 2). There are three known promoters within the p53 gene: two sites upstream of exon 1 producing full-length p53 and one internal site within intron 4 leading to transcription of amino-terminally truncated Δ133p53 (47). Δ40p53 isoforms, which have lost a part of the N-terminal TAD, can be obtained by alternative splicing of exon 2 and alternative initiation of translation at ATG40 (24), while Δ160p53 isoforms, which lack the first 159 residues, arise from translational initiation at ATG160 (48). Alternative splicing of intron 9 generates additional three isoforms, full-length p53, p53β, and p53γ (24). Both 53β and p53γ lack the OD (24). To date, a total of 12 p53 isoforms have been described: p53, p53β, p53γ, Δ40p53α, Δ40p53β, Δ40p53γ, Δ133p53α, Δ133p53β, Δ133p53γ, Δ160p53α, Δ160p53β, and Δ160p53γ (49, 50). While some p53 isoforms exert functions similar to full-length p53, others have antagonizing properties. Δ133p53, for example, inhibits p53-mediated apoptosis and causes cell-cycle arrest at the G2/M checkpoint (47, 50). Δ40p53 isoforms control the development of pluripotent embryonic stem cells into differentiated somatic cells by modulating IGF-1-R levels (51). Very little is known about the clinical role of p53 isoforms and further investigation is needed to determine if they could prove valuable as targets for anti-cancer therapy.

Human p53 protein consists of several domains. The central DNA-binding domain (DBD) (core domain) is shared by most p53 isoforms and binds to response elements of target genes. A large number of p53 mutations occur within this region of the gene (52). The N-terminal transcription–activation domain (TA) is the binding-site for positive (e.g., p300/CBP, TAFII40/60) or negative regulators (e.g., MDM2 and MDMX) of p53 gene transcription (53). The C-terminal oligomerization (CTD) domain is subject to alternative splicing and post-translational modification. The CTD has been shown to influence DNA binding and transcriptional activity of the p53 family members (54).

### p53 regulates cell-cycle, induces apoptosis, and promotes cell differentiation

p53 controls a large number of genes mediating G2/M and G1 cell-cycle arrest, DNA damage recognition, DNA repair, apoptosis, and senescence (25) (Figure 1). Absence of one parental copy of p53 through germline mutation of TP53, a condition called Li–Fraumeni syndrome, leads to development of several tumors, particularly sarcomas and cancers of the breast, brain, and adrenal glands (55, 56). Even in young individuals suffering from this condition multiple malignant tumors may develop. p53 knock-out mice have been shown to be prone to development of various types of malignancies demonstrating the important role of p53 in cancer biology (57). When initiated during the cellular stress response, p53 activates transcription of p21, a cyclin-dependent kinase inhibitor. p21 blocks CDK-1 and -2 leading to cell-cycle arrest at G1 and S phase (58). Since p53 counteracts cell growth and development, it is crucial that p53 function is strictly regulated. The E3 ubiquitin ligase MDM2 blocks p53’s transcriptional activity by binding to the N-terminal TA domain of the protein (59, 60). MDM2 is also capable of inducing the ubiquitin-mediated proteasomal degradation of the tumor suppressor protein (61, 62). In return, p53 positively regulates expression of MDM2. Thereby, it creates an auto-regulatory loop that controls the level of active p53 in the cell (63–65). During the cellular stress response, MDM2 is inhibited by different regulator proteins leading to accumulation of p53 in the cell (66).

Another important upstream regulator of p53 activity is p14ARF, a protein transcribed from an alternate reading frame of the CDKN2A gene locus that also encodes for the tumor suppressor p16INK4a (67, 68). p14ARF is part of the cell’s response to oncogenic activation (69–73). It acts as an inhibitor of MDM2-medited degradation of p53 (74). Therefore, ARF-deficient mice are prone to developing tumors of various entities (75). In a negative feedback loop, ARF promotes degradation of its activator E2F-1 and is suppressed by its downstream target p53 (76, 77).

Primarily, p53 is a transcription factor. It is involved in the intrinsic and extrinsic apoptosis signaling pathways by initiating transcription of functional proteins such as PUMA, Bax, Bid, CD95, and TRAIL-R2 (78). Yet, transcription-independent functions have been described. In the cytosol, p53 induces cell death by forming inhibitory complexes with Bcl-XL and Bcl-2, which leads to the permeabilization of the mitochondrial membrane and cytochrome *c* release (79, 80). Furthermore, cytosolic p53 can activate pro-apoptotic proteins such as Bax and Bak through direct protein–protein interaction (18, 81, 82).

Recently, it was observed that p53 also plays an important role in stem cell biology. In embryonic stem cells, p53 guarantees genetic stability via induction of differentiation (83) while limiting generation of induced pluripotent stem cells and tightly controls reprogramming (84). The cancer stem cell (CSC) hypothesis suggests that every tumor holds a pool of CSCs capable of renewal. They are essential for sustenance and growth of the tumor and respond poorly to conventional chemotherapy (85). CSCs result from either dedifferentiation of somatic cells or mutations in existing stem or progenitor cells (84). Targeting CSCs via activation of p53-linked pathways could trigger cell differentiation. In consequence, malignant cells would be more susceptible to DNA damaging agents and their capacity of self-renewal would be reduced.

In 1997, the cloning of p73 as a new p53 family member was reported, this was followed by the discovery of p63 – the third member of the p53 family (54, 86–89). The protein architecture is highly conserved among the three members of the p53 family (30). The highest degree of sequence homology has been described for the DNA-binding core domain (30). In contrast, the C-terminal domains are diverse and subject to alternative splicing and post-translational modification. Sauer et al. demonstrated that the C-terminal domains influence DNA binding and transcriptional activity (54) and suggested that the diversity of the C-terminal domains of the p53 family influences cell fate decisions and cellular responses that are regulated by the p53 family members (90).

## p63 and Its Isoforms

**Figure 3:**
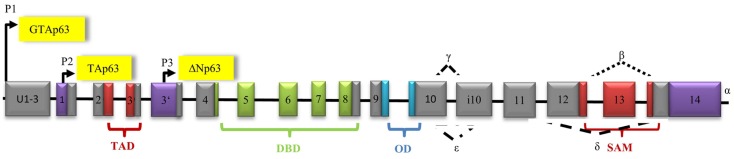
**Architecture of the human p63 gene structure: alternative splicing (α, β, γ, δ, ε), alternative promoters (P1, P2, P3), transactivation domain (TAD), DNA-binding domain (DBD), oligomerization domain (OD), and sterile alpha motif domain (SAM) are indicated**.

The p53 homolog p63 contains three promoters that are known to encode three types of isoforms (91). The first promoter has only recently been discovered by Beyer et al. In response to DNA damage, it leads to activation of human male germ-cell-encoded TAp63 protein, which is specifically expressed in testes and protects the genomic integrity of the male germline (91, 92). The second promoter mediates transcription of TA isoforms, which contain a N-terminal TAD (22% identical with the TAD of p53) followed by a DBD (60% identical with the DBD of p53), an OD (38% identical with the OD of p53), and the sterile alpha motif (SAM) (30). In contrast, there is no SAM in the p53 gene. The third promoter is located between exon 3 and 4. Loss of exons 2 and 3 and incorporation of exon 3′ through the third promoter results in different ΔN isoforms (93). Additionally, alternative splicing at the 3′-terminus leads to the generation of five isoforms (α, β, γ, δ, and ε) and contributes to the variety of proteins (93) Premature transcriptional termination in exon 10 generates isoform ε (94) (Figure 3).

TAp63 is predominantly expressed in oocytes, although it has also been identified in other tissues like epidermis. In TAp63 knock-out mice, a phenotype with ulcers, hair defects, and reduced wound healing can be observed (95).

When first discovered, ΔN isoforms were thought to exclusively repress transcription. But, ΔN isoforms gain their transcriptional activity from two additional TADs within the residue, one located between the OD and the SAM domain and another located in proximity to the proline-rich domain (96, 97). Therefore, they do not only repress functions of the TA isoforms by inhibiting transcription of TA dependent genes but also transactivate their own target genes (98). ΔN63 is found in epidermal cells, in particular (99). Knock-out mice with down-regulated ΔNp63 show severe skin wounds as well as delayed wound healing (100). ΔNp63 expression can be found in multiple tumors, particularly in those with unfavorable prognosis (101). Of importance for clinical use is the fact that ΔNp63α expression is a prognostic marker for poor response to cisplatin chemotherapy in HNSCC (102). However, categorizing ΔNp63 isoforms as proto-oncogenes and TAp63 isoforms as tumor suppressors would be far too simple (103). For instance, diffuse large human B-cell lymphomas do not show enhanced expression of ΔNp63 protein, but overexpression of TAp63 (104, 105).

p63 function is regulated by post-translational modifications that influence p63 protein stability. For example, E3 ligases like Pirh2 and ITCH lead to polyubiquitination and subsequent proteasomal degradation of the protein (106). RNA-binding proteins such as RNPC1, HuR, or PCB1 control stability of p63 by binding AU-, CU-, or U-rich elements in 5′ or 3′ UTRs of p63 mRNA (107–109).

p63 and p53 have common and distinct downstream target genes (110), thereby sharing functions in cell-cycle control and apoptosis (Figure 1). TAp63 causes G1 cell-cycle arrest through transcriptional up-regulation of p21 and p57/Kip2 (111). Furthermore, p63 induces apoptosis via the extrinsic and the intrinsic apoptosis signaling pathway by enhanced expression of Bax, RAD9, DAP3, APAF-1, CD95, TNF-R, or TRAIL-R death receptors (27).

In addition, p63 assumes defined functions within the cell distinct from those of p53. In oocytes, DNA damage directly induces phosphorylation of p63, which leads to oocyte death (112, 113). p63 knock-out mice show a phenotype that is lethal soon after birth. They suffer from significant epithelial abnormalities, concerning skin, glands, teeth, and hair follicles (114). Their limbs are truncated and craniofacial anomalies are characteristic (93, 115). Human heterozygous mutations of p63 result in dysplasia of hair, teeth, digits, sweat glands, and nails (93). Therefore, p63 is essential for epithelial development. Furthermore, in a recent study, D’Aguanno et al. suggested that p63 might be involved in cancer cell metabolism. Colon CSCs showed a higher glycolytic activity when expressing TAp63 instead of ΔNp63 (116). Consistent with these observations, Giacobbe et al. reported that TAp63 isoforms can enhance expression of the mitochondrial glutaminase 2 (GLS2) gene, both in primary cells and in tumor cell lines (117).

Loss of function mutations of p63 are extremely rare in malignancies in contrast to p53 mutations (30) and controversial phenotypes have been described. Development of spontaneous tumors could be found as well as no increase in tumor disposition (111, 118–120). However, alterations in p63 expression patterns play an important role in tumorigenesis (121). In addition, mice heterozygous for mutations in both p53 and p63 (p53+/−; p63+/−) show higher tumor burden in comparison to mice heterozygous for p53 only (118). Knock-down of p63 (p63−/−) can lead to loss of p53 and thereby to cancer development (118). In fact, mice lacking p53 and p63 show increased Ras-mediated sarcoma development (111) and are prone to malignant transformations of embryonic fibroblasts (122). Furthermore, TAp63 has been shown to play an important role in tumor dissemination. Interactions of TGFβ, Ras, and mutant p53 induce formation of a ternary complex of mutant p53, Smads, and the p63 protein, which opposes the anti-metastatic function of p53 (123, 124). TAp63 leads to overexpression of metastasis suppressor genes or microRNAs like DICER1, mir-130b, and integrin recycling genes (116). Mutant p53 can reduce Dicer expression via inhibition of TAp63, thus enabling tumor metastasis (125). The p63 gene controls transcription of the miR-200 family, which regulate CSCs and epithelial–mesenchymal transition (126). ΔNp63α induces miR-205 transcription and regulates epithelial–mesenchymal transition in human bladder cancer cells (127). Therefore, controlling p63 could be a promising approach to control or prevent metastasis in cancer.

## p73 and Its Isoforms

**Figure 4:**
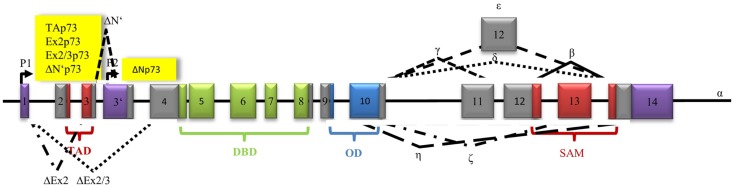
**Architecture of the human p73 gene structure: alternative splicing (α, β, γ, δ, ε, ζ, η), alternative promoters (P1, P2), transactivation domain (TAD), DNA-binding domain (DBD), oligomerization domain (OD), and sterile alpha motif domain (SAM) are indicated**. The P1 promoter generates full-length-proteins with a transactivation domain (TAD), whereas the P2 promoter generates proteins lacking the TAD. Alternative splicing of exon 2 produces Ex2p73 proteins that contain part of the TAD, alternative splicing of exon 2 and 3 produces Ex2/3p73 proteins that have completely lost the TAD. Alternative splicing of exon 3′ generates ΔN′p73.

The p73 gene consists of 15 exons and is located on chromosome 1p36. Like p63, p73 has several TA isoforms containing a specific TAD and ΔN isoforms lacking it (Figure 4). The first promoter, located on exon 1, can induce transcription of several truncated ΔNp73 isoforms. They are either lacking exon 2 or exon 2 and exon 3 (ΔEx2p73 and ΔEx2/3p73). In variant ΔN’p73, exon 3 is substituted by exon 3′. The TAD of p73 is 30% identical to p53. The consecutive p73 DBD shares 63% and the OD 38% identity with p53 (30). The OD is followed by the SAM domain, which is crucial for activating the molecule via tetramerization. At least seven different 3′ terminal splicing variants are known (α, β, γ, δ, ε, ζ, η) (128). Different cell types just express a selection of p73 isoforms (129). Splice variants α and β are rarely expressed in malignant cells (130). Expression of γ, δ, ∈, and θ isoforms has been described in acute myeloid leukemia (AML) and in chronic myeloid leukemia (CML) (131).

There are several molecular mechanisms that regulate p73 function on transcriptional, post-translational, and protein level (32). Enhancers of p73 transcription are p300 (132), E2F-1 (133), CREB-binding protein (CBP) (134), YAP (135), and MM1 (my modulator 1) (136), while MDM2 (137) and c-myc (136) inhibit p73 transcriptional activity. On the post-translational level, p73 activity is reduced by sumoylation by PIAS-1 (138), deacetylation by SIRT (139), threonine phosphorylation by CDK2/CDK-1 (140), neddylation by NEDD8 (141), and conjugation and ubiquitination by Itch (142). In contrast, acetylation by p300 and pCAF (143) or phosphorylation by c-Abl (144), p38MAPk or PKCδ (145) stimulate p73 activity. The RING finger E3 ubiquitin ligase PIR2 selectively ubiquitinates ΔNp73 variants (146). ASPP proteins are also able to regulate p73 function via their poly-C-binding domain (147).

Functions of p73 are diverse. Similarly to its family members p73 plays an important role at different regulatory checkpoints of the cell-cycle. TAp73 induces G1 cell-cycle arrest via enhanced expression of p21 and p57/Kip2 (148). Furthermore, TAp73 represses genes relevant in G2/M-phase like CDC25B and CDC25C (149), Cyclin B1 (150), and Cyclin B2 (149). p73 binds to FLASH and leads to cell-cycle arrest in S-phase (151). As known from p53, DNA damage stimulates p73 to induce apoptosis involving endoplasmic reticulum (ER) stress (152).

Neuronal differentiation is regarded as innate p73 function that is not shared with p53. Phenotype studies of genetically modified mice support this thesis. Most p73 knock-out mice die within the first 4 weeks after birth. They show hippocampal dysgenesis, hydrocephalus *ex vacuo*, atypical social and reproductive behavior, and often suffer from chronic infections (34). Heterozygous mice develop an Alzheimer’s disease-like phenotype with impaired motor and cognitive functions (153, 154). Autopsy revealed accumulation of phosphor-tau positive filaments in the brain and in atrophic neurons (153). TAp73 knock-out mice develop a less severe phenotype characterized by malformations of the hippocampal dentate gyrus (155), whereas ΔNp73 knock-out mice present with reduced neuronal density in the motor cortex, loss of vomeronasal neurons, and Cajal–Retzius cells, as well as choroid plexus atrophy (156, 157). Latest research revealed that TAp73 is a transcriptional activator of the p75 neurotrophin receptor (p75^NTR^), which plays an important role during neurogenesis. TAp73 knock-out mice show reduced levels of p75^NTR^ and suffer from peripheral nerve defect, including myelin thickness and thermal sensitivity (158).

Similarly to p63, p73 executes a set of important functions in tumor metabolism. TAp73 induces the expression of glucose-6-phosphate dehydrogenase (G6PD), which is essential for the oxidative pentose phosphate pathway (159). Cox4il is another p73 target gene relevant in metabolism. Deletion of TAp73 leads to impairment of oxidative phosphorylation via Cox4il. As a result, levels of reactive oxygen species in cells accumulate (160).

p73 is rarely mutated in human cancer (<1%), but overexpression of p73 can be found in several malignancies, for example, in hepatocellular carcinoma (29, 161, 162), neuroblastoma (163), lung cancer (164), prostate cancer (165, 166), urothelial cancer (167), colorectal carcinoma (168), and breast cancer (>40%) (169). Seventy percent of TAp73 knock-out mice or mice heterozygous for p73 suffer from malignant tumors. Colorectal and breast cancer predominantly show an increase in ΔNp73 (170). Overexpression of both, TA and ΔN isoforms, has been detected in thyroid cancer and in chronic B-cell leukemia (171), whereas diminished p73 expression has been reported for pancreatic malignancies (172). p73 heterozygous mice (p73+/−) have an increased probability for the development of spontaneous tumors such as lung adenocarcinoma, lymphomas of the thyme, and hemangiosarcoma (118). Mice heterozygous for mutations in both p53 and p73 (p53+/−; p73+/−) develop a severe disease pattern due to a severe tumor burden and more aggressive tumor dissemination (118).

## p53 Family as a Target of Small Molecules

Large-scale genome sequencing has shown that over half of human malignancies exhibit point mutations in the p53 gene impairing p53 function. Most p53 mutations are missense point mutations located within the DBD. Many of them lead to destabilization of folding of the domain at physiological temperatures and interfere with its DNA-binding ability (173). Certain mutations lead to a gain-of-function of p53 and result in oncogenicity (52, 174, 175). In many other tumors p53, though intact, is inactive following enhanced degradation or reduced activation (176). Loss of wild-type p53 function or gain-of-function is often associated with aggressive tumor growth, poor prognosis, and resistance to chemotherapy. Restoration of p53 function in mice suffering from lymphomas or sarcomas has been shown to induce tumor regression (177, 178). Therefore, restoring wild-type function of p53 holds great promise as a future strategy for cancer treatment.

### Small molecules targeting wild-type p53

**Figure 5:**
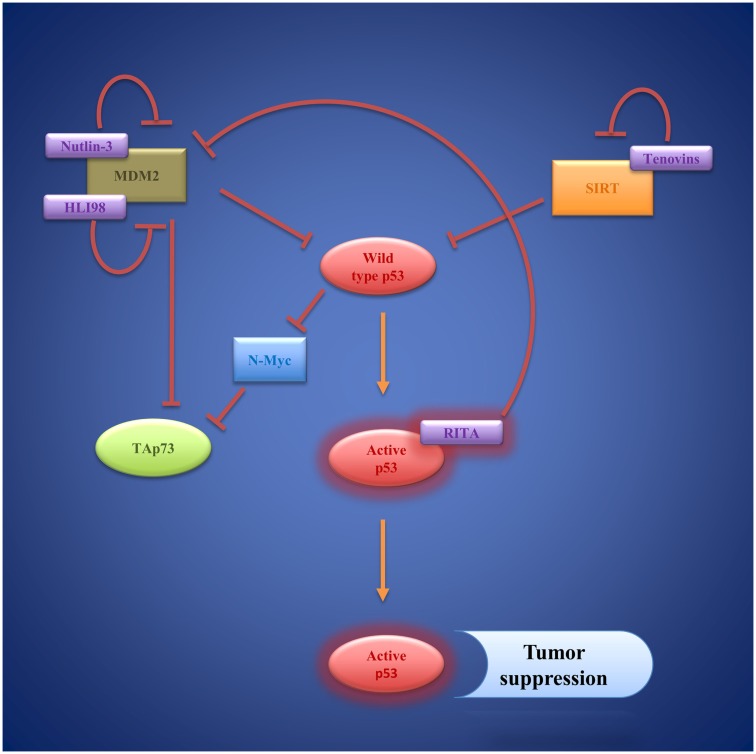
**Wild-type p53 as a target of small molecules: Nutlins, HLI98, and RITA compensate MDM2 inhibition of p53 via inhibition of MDM2**. Tenovins have been identified as SIRT 1 and SIRT 2 inhibitors that indirectly activate p53. Activated p53 induces transcription of genes regulating cell-cycle arrest and apoptosis, resulting in tumor suppression.

To date, a number of small molecules have been identified, which are able to restore wild-type p53 function to cancer cells (Figure 5). The first small molecule inhibitors, which target p53/MDM2-interaction, are Nutlins. Nutlins are a family of three (Nutlin-1, Nutlin-2, Nutlin-3) cis-imidazoline analogs. They occupy the deep hydrophobic pocket of MDM2 that mediates p53 interaction (179). Hence, Nutlins prevent p53 degradation and lead to p53 accumulation and stabilization. There is evidence that Nutlins do not only enhance p53 function but also upregulate p73 in different *in vitro* and *in vivo* settings (180). Nutlin-3a has even proven effective at inducing apoptosis in p53-deficient colorectal carcinoma cells and hepatocellular carcinoma cell lines via activation of p73 (181, 182). A number of preclinical studies, mostly using Nutlin-3 as a therapeutic agent, have been carried out focusing especially on hematological malignancies like AML (183, 184), ALL (185), and B-CLL (186, 187). However, Nutlins are also able to induce apoptosis in other cell lines including ovarian cancer (188), sarcoma (189, 190), as well as glioblastoma (191). Yet, effectiveness of Nutlin therapy ultimately presumes the presence of wild-type p53 and latest findings suggest that it strongly depends on the epigenetic profile of p53 target genes (190, 192). Moreover, Michaelis et al. and Aziz et al. reported on several different cancer cell lines that developed *de novo* p53 mutations and became resistant toward Nutlin-3 mediated apoptosis (193, 194).

Another small molecule that inhibits p53/MDM2 interaction is RITA (reactivation of p53 and induction of tumor cell apoptosis). RITA binds p53 and thereby induces conformational changes within the molecule that prevent MDM2 association (195, 196). In a human head and neck cancer cell line (HNC), RITA was able to restore p53 function contributing to cytotoxicity of cisplatin therapy and leading to apoptosis *in vitro* and *in vivo* (197). The anti-tumoral effect of RITA was also observed in neuroblastoma cell lines (198).

Rational design led to construction of the spiro-oxindole MI-219, which is a highly specific small molecule inhibitor of p53/HDM2-interaction (199). Later, it was discovered that MI-219 does not only induce dissociation of the two molecules but also leads to auto-ubiquitination and degradation of HDM2 (200). MI-219 has been shown to activate p53-dependent pathways, which initiated cell-cycle arrest and apoptosis in a number of cancer cell lines, whereas primary cells remained unaffected by these p53-mediated effects (199). In a preclinical trial, the pharmacological properties of MI-219 were tested and dosages were predicted for use in phase I clinical studies (201).

As an alternative to interfering with p53/MDM2-interaction, degradation of p53 can be prevented by inhibiting the E3 ligase activity of MDM2, and therefore, preventing ubiquitination of p53 (202). A series of 5-deazaflavin derivatives, named HDM2 ligase inhibitor 98 class (HLI98), which bind the C-terminal RING-domain of MDM2, were identified (203–205). Later, it was shown that the nitro group of the molecules is not needed to convey inhibitory function, which led to the synthesis of novel 5-deazaflavin derivatives named MDP compounds (206). While HLI98 and MDP compounds demonstrate an interesting proof of concept, there are still obstacles to overcome in terms of chemical properties such as solubility as well as selectivity for MDM2 (206). Another important question, which needs further attention, is whether inhibition of MDM2 function leads to induction of MDM2 formation via the p53 feedback loop.

The tryptamine JNJ-26854165 (Serdemetan) effectively prevents p53/HDM2 from binding to the proteasome, thereby inhibiting degradation of p53 (207). In acute myeloid and lymphoid leukemia cells, JNJ-26854165 induces apoptosis via p53 by transcription-dependent and -independent pathways (207). A phase I clinical trial assessing safety and dosage of Serdemetan in advance stage and refractory solid tumors showed good bioavailability of the substance and p53 levels in skin biopsies increased. Forty percent of patients showed stable disease, yet in some patients QTc prolongation was observed as an adverse effect (208). However, increased MDM2 levels could render substances like Nutlins, RITA, MDP compounds, and JNJ-26854165 less efficient (209).

SIRT1, a nicotinamide adenine dinucleotide-dependent class III histone deacetylase, deacetylates p53 at Lys382, thereby reducing its activity (210). Hence, blocking SIRT function is a new strategy of restoring p53 function independent of MDM2 (211). Two small molecules, tenovin 1 and the more water-soluble tenovin 6, which block SIRT1 and SIRT2 function efficiently, were discovered by Lain et al. (212). Tenovin 1 was shown to induce apoptosis in cutaneous T-cell lymphoma cells (213). Interestingly, following tenovin 6 treatments cell death was observed in five different colon cancer cell lines independent of their p53 status (214). Also, tenovin 6 activated autophagy-lysosomal pathway genes in chronic lymphocytic leukemia cells without affecting p53 pathways (215). Both findings point toward additional cellular mechanisms mediating the anti-tumor effect of tenovins.

### Small molecules targeting mutant p53

**Figure 6:**
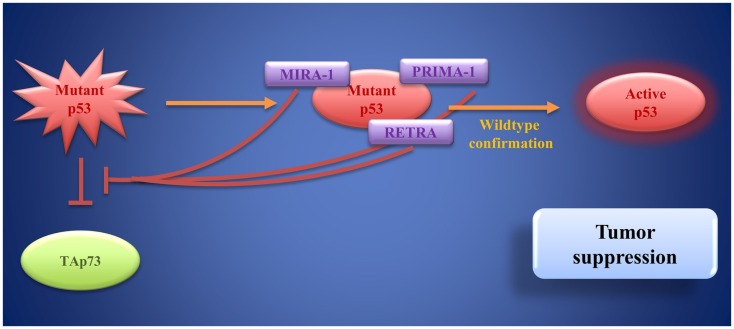
**Mutant p53 as a target of small molecules: PRIMA-1, MIRA-1, and RETRA bind to mutant p53 and restore wild-type p53 function**. Moreover, they block mutant p53-induced inhibition of TAp73. These activities result in tumor suppression.

In tumors that harbor p53 mutations, which often lead to loss of its DNA-binding function, targets for small molecules other than MDM2 are needed. An increasing number of p53 mutations have been described so far. Nevertheless, most mutations cause unfolding of the DBD rendering it unable to bind to target genes for transactivation (216, 217). Therefore, a number of small molecules aiming at restoring and stabilizing the original DBD conformation have been developed (Figure 6). Bykov et al. identified two small molecules by screening a library of low-molecular-weight compounds for substances, which are able to restore wild-type function of mutant p53: PRIMA-1 and MIRA-1 (218, 219). PRIMA-1 (p53 reactivation and induction of massive apoptosis) is a pro-drug (220). The molecule effectively induces apoptosis in bladder cancer cell lines (221). Later, PRIMA-1^MET^ (APR-246), a compound that bears great structural similarities to PRIMA-1, but has higher activity than its predecessor, was discovered (222). Interestingly, PRIMA-1^MET^ can not only restore the pro-apoptotic function of p53 but also of mutant TAp63γ and of TAp73β, while exerting little effect on TAp73α (223). Furthermore, PRIMA-1^MET^ is involved in activating downstream target genes of the p53 family (223–225).

PRIMA-1^MET^ alone and PRIMA-1^MET^ in combination with chemotherapeutic drugs are effective at inducing tumor cell apoptosis *in vivo* (221, 222, 225). Also, a phase one clinical trial using PRIMA-1^MET^ (APR-246) in advanced prostate cancer and hematological malignancies, as well as a phase Ib/II clinical trial using this compound in addition to carboplatin in recurrent high-grade serous ovarian cancer are under way and will offer more insight into the effectiveness and practicability of mutant p53 reactivation (National Cancer Institute: Safety Study of APR-246 in patients with refractory hematologic cancer or prostate cancer; p53 suppressor activation in recurrent high-grade serous ovarian cancer, a Phase Ib/II study of systemic carboplatin combination chemotherapy with or without APR-246).

MIRA-1 (mutant p53 reactivation and induction of rapid apoptosis) is a maleimide-derived molecule and has no structural similarity with PRIMA-1, but it is equally able to restore p53 function leading to cell death via apoptosis with even higher potency than PRIMA-1 (219). By reestablishing its DNA-binding capacity and transcriptional transactivation through p53, MIRA-1 leads to programed cell death in multiple myeloma *in vitro* and in a mouse model (226). To date, little is known about the molecular mechanisms and safety of MIRA-1 treatment and further research is needed before clinical evaluation.

Although PRIMA-1 and MIRA-1 seem to have stabilizing effect on a great variety of p53 mutants, they are not able to restore normal protein configuration to the Phe176 mutant (218). This shows the necessity to test p53 status and to identify the underlying p53 mutations before small molecule treatment (220). In fact, approaches have been made to target distinct mutations. Rational drug design led to the identification of the compound PhiKan083, which stabilizes the Cys 220 p53 mutant and prolongs its half-life, but does not rescue any other p53 mutant (227). PhiKan083 fits into a groove in the defective molecule and induces refolding of the protein (227). In consequence, the melting point of the mutant increases and denaturation is slowed down (227).

CP-31398 was discovered by screening a library of more than 100,000 synthetic compounds for substances that effectively stabilize p53 conformation (228). Initially, CP-31398 was thought to prevent unfolding of wild-type and mutant p53 and increase levels of wild-type p53 by blocking ubiquitination and degradation (229). Yet, further research revealed that it yields a number of p53-independent functions, which mediate its cytotoxic effects (230). In a mouse model of urothelial cancer of the bladder CP-31398 effectively reduced tumor growth and invasion (231).

However, increased p53 activity bares risks for non-cancerous cells that might also be subject to apoptosis and further research is needed to find the adequate dose-response relationship, specific to the compound used (209). In an attempt to identify molecules, which restore p53’s transcriptional activity exclusively in cancer cells holding p53 mutations, reactivation of transcriptional reporter activity (RETRA) was identified by screening compounds from a chemical library (232). Further analysis revealed that RETRA, rather than restoring a functional p53 molecule, leads to an increase in TAp73 levels and to its release from a blocking complex with mutant p53 (232). As mentioned above, p73 can activate various target genes of p53 involved in cell-cycle arrest and apoptosis, thereby mediating tumor cell death (232). *In vivo*, in a xenograft mouse model, tumor growth could be decelerated by intraperitoneal injection of RETRA (232). Although still in the very early stages of development, RETRA opens up new perspectives for p63- and 73-based cancer treatment options.

Moreover, restoring p53 apoptotic function and modulation of p63 and p73 expression is often essential for sensitivity toward chemotherapeutic drugs or radiation, as lack of p53 and unfavorable expression patterns of p63 and p73 can lead to resistance toward treatment in different malignant tumors (233–235). Reconstitution of p53 function or activation of certain p63 and p73 isoforms might allow reducing the dose of cytotoxic drugs while still maintaining their anti-tumor effects. Simultaneously, this would permit to protect normal tissues from side effects of chemotherapy.

However, restoration of wild-type p53 might not be beneficial in all types of tumors. Jackson et al. showed that doxorubicin lead to cell-cycle arrest and senescence instead of cell death in breast cancer expressing wild-type p53, thereby promoting tumor cell survival and resistance to chemotherapy (236). This shows the necessity to elucidate which p53-dependent pathways are favored in certain malignancies before considering small molecule treatment. Novel treatment approaches could lead to the development of substances that selectively activate p53-mediated apoptosis signaling pathways.

## Conclusion

The p53 family plays a central role in cancer development and treatment response. Whereas p53 is often mutated in tumors, p63 and p73 function is preserved, yet altered by different expression patterns of their TA and ΔN isoforms. Increasingly, these expression patterns are evaluated to estimate prognosis and adapt anti-cancer therapy. Nevertheless, the molecular mechanisms regulating the interplay between the different isoforms of the p53 family are only partly understood and are focus of current research. Identifying compounds that interfere with oncogenic signaling induced by certain p63 and p73 isoforms could be a novel approach in anti-cancer therapy.

An increasing number of compounds that re-establish pro-apoptotic p53 function in cancer cells have emerged over the past decade. A variety of small molecules, which aim at increasing p53 function in cancers expressing wild-type p53, have been discovered. Among them are Nutlins, which are already undergoing clinical evaluation, RITA, tenovins, and many others.

In tumors with underlying p53 mutation restoring wild-type activity of p53 has proven more difficult, but nevertheless feasible. PRIMA-1 and MIRA-1 are effective at inducing apoptosis via p53 in tumors that exhibit a great variety of p53 mutations. Yet, there are other small molecules, like PhiKan083, which are more specific and restore wild-type configuration of specific mutants only.

A number of *in vivo* studies and clinical trials have shown synergistic effects of small molecule treatment and chemotherapeutic drugs in a variety of malignancies. Especially cancer cells, which are resistant to chemotherapy due to impaired p53 function, become more susceptible to treatment.

Taking the approaches of p53 reactivation further, there might be new possibilities of targeting CSCs, which are often insusceptible to chemotherapy. Induction of p53 in these cells could lead to activation of pro-apoptotic pathways via differentiation.

## Conflict of Interest Statement

The authors declare that the research was conducted in the absence of any commercial or financial relationships that could be construed as a potential conflict of interest.
